# G-CSF–induced hypercoagulability in two consecutive hematopoietic progenitor cell apheresis procedures: A case report

**DOI:** 10.1097/BS9.0000000000000275

**Published:** 2026-01-28

**Authors:** Yandy Marx Castillo-Aleman, Shinnette Lumame, Charisma Castelo, Shadi Sharif Shamat, Anil Kumar Sarode, Fatma Abdou

**Affiliations:** a Abu Dhabi Stem Cells Center (ADSCC), Abu Dhabi, UAE; b Yas Clinic Khalifa City (YCKC), Abu Dhabi, UAE

## 1. INTRODUCTION

Hematopoietic stem cell transplantation (HSCT) is a key therapeutic approach for treating a range of hematologic and non-hematologic disorders that require CD34^+^ hematopoietic progenitor cells (HPCs) derived from sources such as bone marrow, cord blood, or peripheral blood. In the case of peripheral blood cells harvested through apheresis, mobilization with granulocyte colony-stimulating factor (G-CSF) is commonly employed for both autologous and allogeneic donors.

G-CSF can induce effective HPC mobilization through various mechanisms^1^; however, despite its known benefits, it may trigger adverse reactions, including a hypercoagulable state, by increasing the levels of factor VIII coagulant activity and thrombin generation, placing some donors at increased risk.^2,3^

Herein, we present a case of an allogeneic donor with unexpected G-CSF–induced hypercoagulability, resulting in 2 consecutive unsuccessful HPC apheresis [HPC(A)] procedures.

## 2. MATERIALS AND METHODS

A retrospective data collection was conducted for an allogeneic donor who underwent 3 HPC(A) procedures. Data collected included demographics, mobilization protocol, eligibility and peri-procedure laboratory tests, and apheresis parameters. Intra-apheresis recruitment (IAR) of CD34^+^ cells, captured cells (CC), “crude” collection efficiency (cruCE), and collection efficiency (CE) were calculated according to the equations previously outlined by our group.^4,5^

Informed consent was obtained from the donor and her mother; the study was ethically exempted from review by the ADSCC Institutional Review Board (Ref. MF-5527-2025-02) because it involved retrospective data collection.

## 3. CASE PRESENTATION

A 17-year-old female donor with no significant known medical history was deemed eligible for allogeneic HPC(A) donation. The mobilization protocol required G-CSF (Neupogen^®^; Amgen Inc., Thousand Oaks, California) at 10 µg/kg/d for 5 days. Despite the target yield of 15.0 × 10^6^ CD34^+^ cells/kg (recipient with familial hemophagocytic lymphohistiocytosis), preapheresis parameters exhibited good predictors for successful apheresis, such as significant differences between the donor and recipient weights (55.4 vs 15.5 kg, respectively), and a CD34^+^ cell count of 75 cells/µL.

Although donor screening for the *STXBP2* gene by sequencing, including next-generation sequencing (NGS)-based copy number variant (CNV) analysis, revealed no clinically significant variants, hemoglobin analysis showed hemoglobin A (HbA) 54.3% and hemoglobin S (HbS) 42.7%, with a positive qualitative sickle solubility test consistent with HbS trait.

The HPC(A)-1 procedure was performed using the Spectra Optia^®^ Apheresis System (Terumo BCT, Inc., Lakewood, Colorado) continuous mononuclear cell collection (cMNC) protocol through peripheral venous accesses with anticoagulant citrate dextrose solution A (ACD-A) at a starting anticoagulant-inlet ratio of 1:12. Shortly after initiation, clotting was observed in the nonanticoagulated inlet line.

Despite changing the drawing access 2 times and reducing the anticoagulant-inlet ratio up to 1:8, clotting was extended to the kit, thereby preventing further collection. Thus, the apheresis team expected a yield of 7.89 × 10^6^ CD34^+^ cells/kg (considering a CE_2_ of 35%), so decided to re-collect with another kit. HPC(A)-2 commenced 40 minutes later; despite an average anticoagulant-inlet ratio of 1:10, this run was also aborted owing to a kit massive clotting, whereas the predicted yield was calculated as 12.95 × 10^6^ CD34^+^ cells/kg summed both aphereses. However, the combined yield was significantly lower than expected (2.56 × 10^6^ CD34^+^ cells/kg), with a CE_1_ of only 6.92%. No dehydration, electrolyte imbalance, or remarkable symptoms or signs were noted during these procedures. Postprocedure investigations showed a shortened activated partial thromboplastin time and international normalized ratio (INR) without thrombocytosis.

The following day, HPC(A)-3 was started with a preapheresis CD34^+^ cell count of 82 cells/µL, using a femoral Medcomp^®^ Duo-Flox^®^ Side by Side double-lumen catheter (Medical Components, Inc., Harleysville, Pennsylvania) with heparin combined with ACD-A (10 units/mL of citrate). With these new settings, the procedure was uneventful, with a greater yield of 35.23 × 10^6^ cells/kg, CE_1_ of 77.53%, and an IAR of CD34^+^ cells of 501.69 × 10^6^, vs 106.24 × 10^6^ of both HPC(A)-1/2 together.

**Figure 1.**
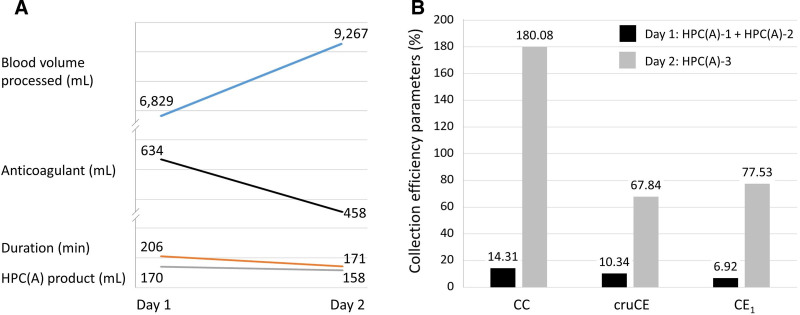
The pre- and postapheresis blood cell counts, procedural parameters, and collection outcomes for days 1 [HPC(A)-1 and HPC(A)-2] and 2 [HPC(A)-3], respectively. (A) Procedural parameters and (B) collection outcomes of CD34^+^ cells. CC = captured cells, CE_1_ = collection efficiency 1, cruCE = crude collection efficiency, HPC(A) = CD34^+^ hematopoietic progenitor cell apheresis.

Table 1 and Figure 1 summarize the pre- and postapheresis blood cell counts, procedural parameters, and collection outcomes for days 1 [HPC(A)-1 and HPC(A)-2] and 2 [HPC(A)-3], respectively.

**Table 1:** Pre- and postapheresis blood cell counts and coagulation profiles.

Parameter	Reference range	Day 1		Day 2	
		Pre HPC(A)-1	Post HPC(A)-2	Pre HPC(A)-3	Post HPC(A)-3
White blood cells (×10^3^/µL)	4.50–13.00	21.9	56.41	21.93	69.76
Neutrophils (×10^3^/µL)	2.00–7.00	16.38	48.32	16.68	63.79
Lymphocytes (×10^3^/µL)	1.00–3.00	3.46	3.96	3.27	3.31
Monocytes (×10^3^/µL)	0.20–1.00	1.70	3.79	1.62	2.35
Platelets (×10^3^/µL)	140–400	235	151	168	140
CD34^+^ cells (/µL)	≥10*	75	93	82	70
aPTT (s)	25.1–36.5	33.2†	22.8	NA	33.2
PT (s)	9.5–13.8	11.5†	12.3	NA	13.8
PT control (s)	10.0–13.0	10.3†	13.0	NA	13.0
INR (ratio)	0.8–1.1	1.0†	1.1	NA	1.2

aPTT = activated partial thromboplastin time, HPC(A) = CD34^+^ hematopoietic progenitor cell apheresis, INR = international normalized ratio, NA = not available, PT = prothrombin time.

* Post-mobilization.

† Before mobilization.

## 4. DISCUSSION

The complications observed during HPC(A)-1 and HPC(A)-2 may represent G-CSF–induced hypercoagulability in this donor with HbS trait. The role of G-CSF in inducing hypercoagulable states after mobilization and collection has been well documented.^2,3,6^ Moreover, the extracorporeal exposure of blood to plastic surfaces may facilitate the adsorption of clotting factors, platelet aggregation, and the adherence of various cells.^7^

Kearney et al^8^ recently reported that the hypercoagulable states commonly observed in patients with sickle cell disease can be resolved by decreasing the anticoagulant-inlet ratio using ACD-A alone, which was futile in our case.

Despite these G-CSF effects, we believe that difficult peripheral venous access that leads to continuous interruption of extracorporeal circulation with prolonged pumps and centrifuge stops could also contribute to platelet activation and coagulation cascade. This was resolved by using a femoral central line during the HPC(A)-3 procedure, along with additional anticoagulation using heparin. In this regard, we concur with Prisciandaro et al,^9^ who stated that central venous access should be considered a last resort for healthy HPC(A) donors.

Additionally, Merter et al^10^ reported that heparin plus citrate anticoagulation enhances HPC(A) collection and increases CE, which may explain the greater IAR pool of CD34^+^ cells observed in HPC(A)-3. Although Gereklioglu et al^11^ reported that G-CSF is generally safe in donors with HbS trait who achieved adequate HPC(A) yields and showed no statistically significant differences in early or late side effects compared with donors without hemoglobinopathies, a G-CSF hypercoagulability risk-stratification model should be implemented in routine practice. This model should consider not only underlying diseases but also personal or family history of venous thromboembolism, inherited or acquired coagulation disorders, age, obesity, use of oral contraceptives or hormone replacement therapy, and other chronic inflammatory conditions. In addition to optimizing venous access and anticoagulation, as described here, further precautions may include confirming HbS status before mobilization, ensuring adequate hydration and oxygenation, using lower G-CSF doses (eg, 5 µg/kg twice daily instead of 10 µg/kg once daily), and maintaining close clinical and laboratory monitoring during and after mobilization and HPC(A) collection.

## 5. CONCLUSIONS

For apheresis donors experiencing G-CSF-induced hypercoagulability, special considerations of adequate venous access and enhanced anticoagulation protocols are recommended. Moreover, implementing a G-CSF hypercoagulability risk-stratification model, with emphasis on close clinical and laboratory monitoring during and after mobilization and HPC(A) collection, may help tailor strategies to ensure successful HPC harvesting in challenging scenarios.

## ACKNOWLEDGMENTS

The authors gratefully acknowledge the team of physicians, nurses, laboratory scientists, and other stakeholders of our Abu Dhabi Bone Marrow Transplant (AD-BMT) Program for their support of the apheresis services.

## AUTHOR CONTRIBUTIONS

Y.M.C.-A. performed conceptualization, data curation, formal analysis, investigation, validation, visualization, writing-original draft, writing-review and editing; S.L., C.C., S.S.S., A.K.S., and F.A. performed data curation, validation, and writing-review and editing. All authors have agreed to be personally accountable for their contributions.
